# Exploring visitor perception and environmental element relationships in rock carving landscapes through random forest analysis

**DOI:** 10.1371/journal.pone.0326302

**Published:** 2025-07-01

**Authors:** Wenxue Liu, Huan Li, Hao Sun, Qiaoyun Sun, Yishu Xia

**Affiliations:** 1 School of Landscape Architecture and Art, Fujian Agriculture and Forestry University, Fuzhou, China; 2 School of Computer Science and Engineering, Macau University of Science and Technology, Macau, China; 3 School of Architecture and Urban Planning, Shenzhen University, Shenzhen, China; 4 School of Design Art, Changsha University of Science & Technology, Changsha, China; Tel Aviv university, ISRAEL

## Abstract

As a typical representative of cultural landscapes, the perceptual assessment of rock carvings encompasses not only the artistry of the carvings themselves but also the multi-dimensional impact of the surrounding environment on tourists. However, existing studies primarily focus on evaluating the artistic value of rock carvings while overlooking the influence of the environment on visitor experience. Taking Yeshan Spring and Autumn Garden as a case study, this research collected image data and visitor perception surveys to establish key perception indicators and employed the random forest method to analyze the importance of environmental factors and their association with visitor perception. The findings indicate that uniqueness and comfort are the key factors shaping visitor experiences, with rock carvings serving as the core anchors attracting visitors. Additionally, vegetation elements—particularly middle vegetation—demonstrated significant potential in influencing and enhancing visitor perception. This study preliminarily reveals the complex relationships between environmental elements and visitor perception and highlights the nonlinear contribution of the synergy among garden elements to improving visitor experience. Following the “one garden, one policy” design principle, this study emphasizes the importance of tailoring conservation and development strategies based on the unique natural conditions, historical and cultural background, and social needs of each garden. It underscores the necessity of optimizing the synergy among garden elements as an effective approach to enhancing visitor perception. By integrating quantitative techniques, this research constructs a more comprehensive evaluation system, providing a valuable reference for cultural heritage preservation and ecological sustainability.

## 1. Introduction

Rock carving is a unique form of ancient Chinese stone carving art, specifically referring to inscriptions, sculptures, or rock paintings carved into the rock faces of mountains [1]. This art form originated as a method of recording historical events and flourished during the Wei, Jin, and Northern and Southern Dynasties (220–589 CE). It continued to evolve through the Tang (618–907 CE), Song (960–1279 CE), Yuan (1271–1368 CE), Ming (1368–1644 CE), Qing (1644–1912 CE), and Republican periods (1912–1949 CE), leaving behind a wealth of invaluable works [2,3]. Abroad, Clottes, Jean points out in his book World Rock Art that rock art sites are a globally shared cultural phenomenon spanning all five continents and tens of thousands of years of human history [4]. These artistic creations not only reflect the complex systems of thought across different regions but also testify to the fundamental unity of the human spiritual world. Through the act of carving, humans have inscribed cultural elements such as beliefs, symbols of power, and collective memory onto rock surfaces. These rock carvings are both products of human social activities (i.e., “taskscapes”) and harmoniously integrated with the natural topography (i.e., “landscapes”), embodying a profound interaction between humans and nature [5]. The content of these engravings is extensive, covering historical events, cultural figures, poetry and literature, as well as architectural works, collectively presenting a comprehensive picture of societal life and cultural values at that time. Therefore, in understanding the relationship between human activities and the environment, it is essential to place everyday human practices—such as farming and dwelling—within a broader spatiotemporal framework; that is, to regard them as part of an continuously evolving world over time [6,7].

According to the data from the eight batches of national key cultural heritage protection sites, rock carvings are distributed across 70 locations [8]. However, their spatial distribution exhibits characteristics of “large aggregation, small dispersion,” with a broad but uneven coverage. Although the rock carving techniques enjoy local renown, their profound historical heritage and unique cultural connotations have not been fully explored or passed down, resulting in low visitor awareness of rock carving culture and making it difficult to achieve lasting and high-impact cultural tourism development [9,10]. In addition, due to the low regional concentration of rock carvings, inadvertent damage occurs from time to time. As such, enhancing the social visibility of rock carvings has become a critical task. Christopher Tilley and scholars such as Bender have pointed out that landscape is not merely a naturally existing space, but also a cultural product co-constructed through people’s daily experiences and interactions. Public perception involves not only physiological responses, but is also deeply rooted in specific cultural contexts and worldviews [5,7]. Research indicates that, from the public’s perspective, the integration of traditional Chinese culture with modern design holds immense potential for both cultural heritage preservation and innovation [11]. Based on this, our paper focuses on how to achieve innovative expressions of rock carvings through modern artistic means to promote their cultural dissemination and preservation.

The carving techniques and content of rock inscriptions reflect a high level of artistic value [12]. Innovative transformation emphasizes “adapting the valuable elements of traditional culture to meet the needs of the times, endowing them with new contemporary meanings and forms of expression, thus invigorating their vitality” [13]. This suggests that the culture of rock inscriptions can be reinterpreted and preserved through modern media and technologies [14]. Specifically, this can be achieved by using material products as a medium to record and reshape the culture of rock inscriptions, integrating traditional elements into contemporary art design for innovative research [15]. For instance, by integrating the artistic features of rock inscriptions into modern design through visual communication elements such as overall composition, type of script, arrangement, and spatial relationships [16]. At the same time, based on the principles of cultural significance, practicality, and innovation, we explore the integration of rock carvings with cultural and creative products, and tap into their potential applications in modern design [17].

However, existing rock inscriptions face numerous challenges related to inheritance and development [18]. Firstly, the materials used for rock inscriptions, such as granite and limestone, have been exposed to natural environmental elements for a long time. Factors like weathering, erosion from rainwater, and friction with trees have caused varying degrees of damage to the clarity of the carvings, leading to an overall degradation [19–21]. Secondly, the public’s awareness of rock inscriptions is relatively shallow, and there is a lack of protection consciousness. Some individuals engage in actions such as “restoring” or “repairing” the carvings, which can cause irreversible damage [22–24]. Therefore, the inheritance and development of rock inscriptions is a critical issue that contemporary society must address.

This study adopts a public perspective to focus on the perceptual mechanisms of monumental rock carvings, systematically evaluating their multidimensional perceptual characteristics by analyzing the multisensory interactions (including vision, hearing, and touch) experienced by the public in tourism contexts. The research guides the formation of spontaneous perceptual evaluations by the public. Combining questionnaire-based scoring with image element recognition technology, the study extracts and quantifies key visual elements (such as vegetation distribution, architectural form, pathway design) and perceptual indicators (such as aesthetic value, spatial comfort, and artistic expressiveness) to comprehensively analyze the natural attributes and artistic value of the rock carvings. Integrating machine learning algorithms, this study explores the complex nonlinear relationships between visual elements and perceptual indicators, uncovering their intrinsic connections and mechanisms in the perceptual evaluation of rock carvings. This research framework not only provides a scientific basis for understanding the cultural connotations of monumental rock carvings but also lays a theoretical foundation for innovative practices in cultural heritage conservation and dissemination.

## 2. Literature review

### 2.1 Landscape perception, art, and design

Landscape, as a complex structure, evolves from the transformation of a piece of land and has gradually developed into a classic interdisciplinary concept [25]. It not only reflects social realities but also carries historical heritage and cultural accumulation, becoming an entity that integrates both spatial and cultural attributes [26]. Landscape perception is immersive and multimodal, capable of simultaneously providing both central and peripheral information. However, the volume of information often exceeds human processing capacity, sometimes leading to redundancy or contradictions. Nevertheless, the core significance of landscape perception lies in its action-oriented nature—it stimulates purposeful human behavior and, through its unique atmosphere, aesthetic qualities, and systematic characteristics, profoundly influences interactions between people and their environment [27]. Art and design are not only central elements of landscape design but also serve as crucial bridges for connecting public mental well-being, enhancing the quality of leisure experiences, and preserving cultural values [28].

Landscape art and design are artistic crafts centered on formal aesthetics, aiming to address environmental processes and functional challenges through creative approaches [29]. Dee (2012) introduced the concept of “frugal aesthetics,” providing an operational framework grounded in humility and originality. This concept emphasizes achieving a balance between artistic value and ecological objectives in a minimalist and efficient manner [30]. Landscape design (LD) is fundamentally a form of artificial ecological design dedicated to integrating the harmony and beauty of natural environments into the expression of environmental art [31]. It not only focuses on maintaining ecological integrity and promoting the healthy circulation of ecosystems but also emphasizes the efficient use of resources to avoid unnecessary waste [32]. Additionally, landscape design deeply incorporates rich cultural connotations and aesthetic values, reinforcing artistic expression and the transmission of beauty, thereby serving as a bridge between human emotions and the natural environment [33].

By combining natural and artificial landscapes, landscape design fully embodies the designer’s subjective intent and creative expression, striving to achieve harmonious coexistence between humans and nature while meeting environmental needs as much as possible [31]. Therefore, landscape art and design are not merely visual presentations but also function as mediums for social interaction and psychological perception [34]. Through an in-depth analysis of the artistic expressions of landscapes and their psychological and behavioral impacts on humans, we can better understand how to utilize landscape resources to enhance public well-being, strengthen cultural identity, and advance sustainable development goals.

### 2.2 Rock carving art, nature, and psychological perception

In recent years, an increasing number of studies have shown that rock carving art not only conveys cultural connotations to visitors but also plays a crucial role in enhancing psychological comfort [3,35]. As an innovative fusion of human artistic creation and the natural environment, rock carvings serve not only as carriers of historical memory but also as essential links connecting art, design, and experience [36]. They provide the public with multidimensional perspectives and profound experiential engagement in tourism [3].

Research on rock carving landscapes has extended into multiple disciplines, including psychology, tourism studies, and landscape architecture [37,38]. The unique artistic form of rock carvings, seamlessly integrated with the natural environment, not only evokes a sense of awe toward historical and cultural heritage but also stimulates positive psychological responses through sensory experiences such as vision and touch. This, in turn, enhances visitors’ physical and mental well-being and provides a sense of spiritual fulfillment. Therefore, rock carving art is not only a valuable cultural heritage but also a cultural medium that fosters harmony between humans and nature, warranting further exploration and conservation.

The Chinese government, through laws and regulations such as the ‘Law of the People’s Republic of China on the Protection of Cultural Relics’, has established a strict protection framework at the national level, emphasizing the principle of “protection first” [39]. This provides a solid legal guarantee for China’s rich rock carving heritage. At the same time, local governments at all levels are also implementing specific protection measures, such as the Chongqing municipal government’s formulation of special protection management regulations for the Dazu Rock Carvings, ensuring effective management and maintenance of the heritage sites [40]. In addition, with the development of digital technology, China’s heritage interpretation strategies continue to innovate, for example, by creating digital museums and virtual reality experiences to showcase rock carving art, making it more convenient for the public to access these precious cultural heritages [41]. This comprehensive management approach not only promotes understanding and appreciation of rock carving art but also lays the foundation for the protection and inheritance of these non-renewable cultural resources for future generations. Therefore, rock carving art not only holds profound significance as a cultural heritage but its management model and protection measures are also key to achieving long-term sustainable development.

### 2.3 Evaluation of rock carvings and visitor experience

The evaluation of rock carvings encompasses diverse aspects. Traditional methods primarily rely on expert scoring, which is highly subjective. Although emerging technologies such as infrared detection [42] and drone observation [43] can provide high-precision data, they are costly and limited to specific time points, making it difficult to capture long-term dynamic changes. The most direct approach to assessing visitors’ on-site perceptions is real-time rating [44].

The semantic differential method, a psychological measurement tool, effectively transforms qualitative perceptions into quantitative data, providing a scientific basis for evaluating visitor experiences [45]. This method utilizes pairs of opposing adjectives (e.g., “elegant—rough” or “tranquil—noisy”), requiring visitors to rate the characteristics of rock carvings based on their subjective feelings [46]. By incorporating both aesthetic values and psychological responses, this approach offers a more comprehensive assessment [47].

In the comprehensive evaluation of rock carving aesthetics, previous studies have attempted to integrate multiple indicator systems [48,49], though most rely on linear regression models. While these models provide a reference framework for evaluating natural environment indicators, the complex and nonlinear relationship between visitor experience and landscape value suggests that traditional linear approaches may not fully capture underlying patterns [50]. Therefore, incorporating nonlinear or multivariate statistical methods can better characterize these interactions, offering deeper insights into the relationship between visitor experience and landscape value. Such advancements can provide more targeted guidance for the conservation and sustainable development of rock carvings.

### 2.4 Research questions and contributions

This study aims to explore the influence mechanisms of micro-environmental elements in rock carving scenic areas (e.g., sky, hydrology, vegetation, architecture) on visitor perception and evaluation. Specifically, it focuses on the relationship between environmental characteristics (e.g., vegetation richness, uniqueness, biodiversity) and psychological responses (e.g., comfort, aesthetic appreciation, sense of security). By employing Principal Component Analysis (PCA) to extract key influencing factors, this research seeks to uncover how these factors collectively shape visitors’ overall experiences. The core research questions include: 1) Which specific elements of rock carvings and their surrounding environment have a significant impact on visitors’ psychological perceptions? 2) What are the relationships and underlying mechanisms between environmental characteristics and psychological responses? 3) How can scenic area design be optimized to enhance the overall visitor experience while ensuring the sustainable conservation of cultural heritage?

The contributions of this study are threefold. First, at the theoretical level, it constructs a quantitative analytical framework for examining the relationship between micro-environmental characteristics of rock carving scenic areas and visitor psychological perceptions. By categorizing perception indicators into environmental characteristics (e.g., vegetation richness, uniqueness) and psychological responses (e.g., comfort, aesthetic appreciation, sense of security), this study systematically reveals the multidimensional influence mechanisms of rock carvings on visitor attraction, providing a more comprehensive perspective for understanding visitor experiences. Second, at the practical level, the findings provide scientific guidance for the planning and management of rock carving scenic areas. The results can assist decision-makers in optimizing resource allocation and achieving a balanced approach between ecological conservation and tourism development, thereby enhancing the overall management and sustainable development of these sites. Finally, by integrating natural and cultural elements, this study expands the research domain of cultural heritage conservation and visitor experience optimization, offering valuable insights for both academic research and practical applications.

## 3. Methods

### 3.1 Study area

This case study selected Fuzhou, located in southeastern China’s Fujian Province, a city with a rich heritage that traces back to the establishment of the first walled city in Fujian, known as Yecheng, situated at the northern end of Fuzhou’s urban central axis within the Yishan historical and scenic area. As an important birthplace of Mindu culture, this area has witnessed more than 2200 years of evolution and development of this historic and cultural city [51]. Fuzhou boasts a rich heritage of rock carving cultural relics, with a total of 171 rock carving sites and 57 designated cultural heritage protection units, ranking among the top in the country [52]. The city’s rock carvings are primarily concentrated in Gushan (Drum Mountain), Wushi Mountain, Yushan, Yeshan, and suburban areas such as Xiangshan. For this study, Yeshan Chunqiu Garden was chosen as the research site based on two key considerations. First, the area holds significant historical and cultural value and enjoys high public recognition, attracting a large number of visitors, making it one of Fuzhou’s most important cultural tourism destinations. Second, the rock carvings within Yeshan Chunqiu Garden are well-preserved, abundant in number, and were included in the ninth batch of provincial-level protected cultural heritage sites in 2018, underscoring their crucial role in cultural heritage conservation. These characteristics make Yeshan Chunqiu Garden an ideal case for evaluating the functionality of rock carving scenic areas and visitor experiences.

### 3.2 Independent and outcome variables

#### 3.2.1 Tourist perception variables of rock carvings.

Tourist perception variables of rock carvings can be categorized into environmental characteristics [3] and psychological perception [53]. These two types of variables together form a comprehensive evaluation system for the perception of rock carvings and their surrounding environment. The environmental characteristics variables include vegetation richness, uniqueness, integration of carvings with the surrounding environment, architectural harmony with carvings, number of carvings, and biodiversity. These indicators reflect both the natural ecological and cultural landscape attributes of the scenic area. Such objective factors not only directly shape tourists’ visual experience but also indirectly influence their perception of the cultural significance of rock carvings. The psychological perception variables focus on tourists’ subjective emotional responses and psychological experiences within the scenic area. These include comfort, aesthetic appreciation, sense of security, artistic value, historical educational significance, and overall attraction. This set of variables aims to deepen the understanding of tourists’ psychological needs and provide key insights for enhancing their satisfaction and psychological well-being. Together, these two categories of variables—objective environmental features and subjective psychological responses—offer a holistic assessment of tourists’ perceptions and satisfaction with rock carvings. This dual-dimensional framework provides a systematic analytical basis for further research and practical applications in scenic area management and cultural heritage conservation.

#### 3.2.2 Micro-environmental variables of rock carvings.

**Fig 1:**
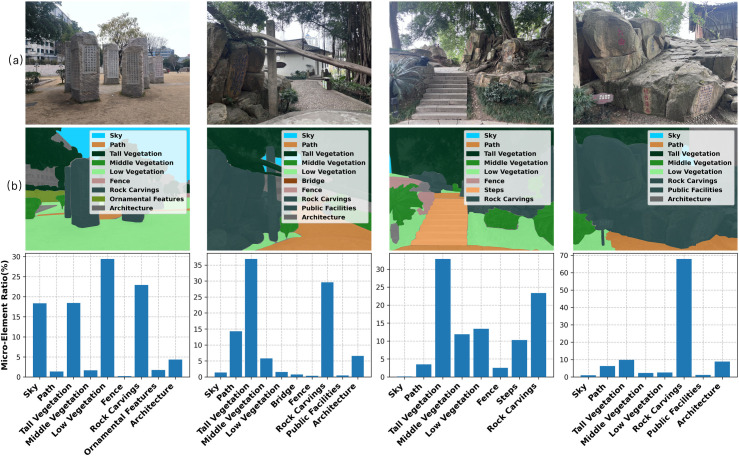
(a) Original image samples (b) Image semantic segmentation and quantitative results.

From the perspective of micro-environmental elements in scenic areas, this study employs image semantic segmentation technology to systematically analyze the environmental characteristics of rock carving sites. By processing on-site image data, the overall environment is quantified and classified, extracting key factors that influence tourists’ perceptions (Fig 1). These micro-environmental variables are primarily divided into two categories: natural elements and artificial elements(Table 1). Natural elements include sky, hydrology, and vegetation, representing the natural landscape features of the site. Artificial elements encompass paths, buildings, and facilities, reflecting human-designed structures within the scenic area. Together, these elements form the micro-environmental system of rock carving sites, providing essential data support for in-depth analysis of tourist experiences.

**Table 1:** Image segmentation elements annotation table.

Element	Annotation
Sky	Visible sky elements in the image.
Water Features	Streams, lakes, ponds, seas, springs, and waterfalls.
Path	Pedestrian path, recreational trail, highway route, etc.
Tall Vegetation	Large and medium-sized trees (9-12m); small trees and ornamental plants (4.5-6m), tall shrubs (3-4.5m)
Middle Vegetation	Medium shrubs (1-2m); low shrubs (30 cm-1m)
Low Vegetation	Ground cover plants (15–30 cm); lawn (<15 cm)
Bridge	Slab bridge, winding bridge, arch bridge, pavilion bridge, etc.
Pavilion	A small structure in a garden or garden for rest and viewing.
Plaza	An open public space for gatherings, leisure, or events.
Fence	A barrier used to demarcate areas or provide safety.
Steps	Stair structures connecting different ground levels for easy access.
Rock Carvings	Inscriptions, patterns, or artworks carved into stone.
Ornamental Features	Decorative designs in gardens or buildings, such as sculptures or fountains.
Public Facilities	Service facilities provided for public convenience, such as benches or restrooms.
Architecture	The design, construction, and artistic style of buildings.

#### 3.2.3 Dependent variable: Tourist experience evaluation.

The tourist evaluation data was collected on-site through three rounds of data collection from October 1 to October 3, 2024. A total of 116 images were captured, and corresponding questionnaires were distributed (S1 Appendix). After filtering the dataset based on image completeness, 96 valid images were selected as evaluation samples. In the questionnaire design, this study employed the Semantic Differential (SD) method [54]. The SD method allows for the quantitative measurement of public perception of landscapes by converting qualitative impressions into numerical values. This makes it a widely used tool in urban public spaces [55], landscape environments [56], and architectural planning [57]. The data collection process included the following steps: 1) Identifying key environmental features of the target location and ensuring the completeness of the landscape image dataset. 2) Performing manual semantic segmentation annotation using the Labelme tool. Distinct landscape elements (e.g., tall vegetation, architecture, water Features, rock carvings, and pathways) were outlined pixel-wise, and annotations were saved in JSON format, including object categories, boundary coordinates, and fill colors. Annotation accuracy was verified through script-based visualization. 3) Conducting a questionnaire survey, in which 30 participants were asked to evaluate images taken at the location. Respondents rated the images using a three-point scale (−1, 0, + 1) [58], representing negative, neutral, and positive perceptions, respectively. 4) Collecting and organizing quantitative data from the questionnaires to support subsequent analysis and research.

**Fig 2:**
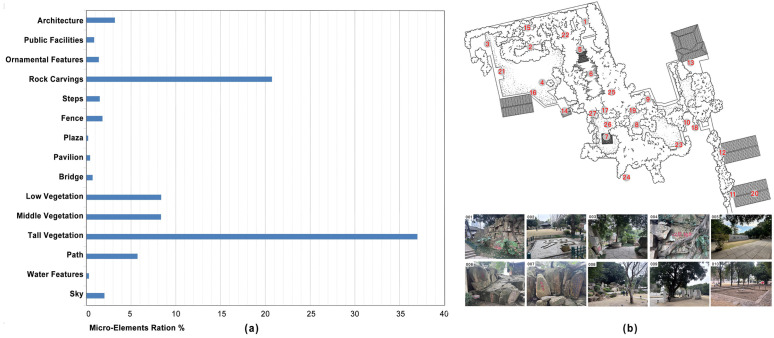
(a) Proportion of landscape elements in images; (b) Shooting and evaluation locations of 27 fixed-point positions.

This study focused on visitors to Yeshan Chunqiu Garden, employing a method that combined fixed-point observation with image evaluation. Although a larger sample size is generally preferable, practical limitations often come into play. “Social Survey Methods” sets a minimum standard for the required sample size at 25, with a margin of error of ±20% [59]. In psychological research, a sample size exceeding 30 is considered a large-sample experiment, as it is believed to offer higher reliability [60]. As illustrated in the research methodology outlined in Fig 2(b), 30 participants conducted on-site assessments at 27 fixed observation points, simultaneously completing image rating tasks corresponding to the in-situ landscape (an average of 27 questionnaires per participant). Collectively, they assessed a total of 96 sets of landscape images, resulting in 810 valid data entries.

The demographic characteristics of the participants are as follows: there were 14 male and 16 female participants, with ages ranging from 20 to 56 years, covering a wide age range and helping to capture perspectives from different age groups. In terms of occupation, the majority of participants were students, including both undergraduate and graduate students, followed by company employees. Local residents and students showed higher levels of engagement, indicating strong interest in the topic or greater flexibility in their schedules. Regarding educational background, the overall education level of the participants was relatively high, with most holding at least a bachelor’s degree, and some possessing master’s or even doctoral degrees. This suggests that the participants had strong analytical abilities and were likely able to understand and respond to complex questions in a thoughtful manner.

#### 3.2.4 Statistical analysis.

In the statistical analysis of this study, Principal Component Analysis (PCA) from the Scikit-Learn scientific computing library in Python was utilized to reduce the dimensionality of 12 interrelated evaluation factors through an orthogonal transformation mechanism. This approach effectively extracted key information from the data and integrated the most significant principal components into a comprehensive Core Perception Index, serving as the critical dependent variable for quantifying and characterizing overall perception attributes. The dataset, consisting of 96 samples, was partitioned using cross-validation, with 85% designated as the training set and 15% as the test set. The study employed two machine learning algorithms: Support Vector Machine (SVM) and Random Forest. SVM is a supervised learning model designed to identify the optimal hyperplane that maximizes the margin between samples [61]. In contrast, Random Forest constructs and trains multiple independent decision trees in parallel, leveraging ensemble learning to optimize classification and regression tasks [62].

Model evaluation was conducted using metrics such as Accuracy, Precision, Recall, and F1-Score. Specifically, the macro F1-Score was employed to calculate the averaged precision and recall across all categories, ensuring a comprehensive and balanced performance assessment.


$$Precision=\frac{TP}{TP+FP}$$ (1)

$$Recall=\frac{TP}{TP+FN}$$ (2)

F1−Score=2·(precision·recallprecision+recall) (3)

$$Macro\;F1-Score\;=\;\frac{1}{C}\sum_{i=1}^{C}{F1-Score}_{i}$$ (4)

$$Weighted\;F1-Score\;=\;\frac{\sum_{i=1}^{C}w_{i}\cdot {F1-Score}_{i}}{\sum_{i=1}^{C}w_{i}}$$ (5)

In Equations (1) to (5): TP represents the number of samples that are actually positive and correctly predicted as positive. FP represents the number of samples that are actually negative but incorrectly predicted as positive. FN represents the number of samples that are actually positive but incorrectly predicted as negative. ${F1-Score}_{i}\;$ is the harmonic mean of precision and recall for class *i*. C  denotes the total number of classes. $w_{i}\;$ represents the weight of class *i* within the dataset. Macro F1−Score is obtained by calculating the F1−Score for each class individually and then taking the arithmetic mean. Weighted F1−Score  is the weighted average of the F1−score across all classes, considering their respective proportions in the dataset.

## 4. Results

### 4.1 Descriptive statistics

Based on the designated tour route of Yeshan Chunqiu Garden, we conducted fixed-point data collection. After filtering out highly redundant image materials, we ensured comprehensive coverage of all visual elements within the garden, ultimately selecting 96 representative images. To further analyze the content of these images, we employed advanced image semantic segmentation techniques to meticulously segment and categorize various landscape elements. According to the data collection and analysis results presented in Fig 2(a), the overall spatial composition of Yeshan Chunqiu Garden is predominantly characterized by vegetation elements, with tall vegetation accounting for the largest proportion among all visual components. Rock carvings represent another key element, ranking second in terms of proportion. Meanwhile, although middle and low vegetation occupy a smaller share, they still hold a noteworthy presence in the overall landscape composition.

### 4.2 Selection and description of indicators

Based on the results of Principal Component Analysis (PCA), the 12 feature variables were categorized into Natural Environment Perception Indicators (E1–E6) and Psychological Experience Perception Indicators (E7–E12) (Table 2). The data revealed that, among the natural environment perception indicators, uniqueness exhibited the highest level of importance, followed by vegetation richness. This suggests that these two physical environmental elements are the primary factors attracting visitors during their tour of Yeshan Chunqiu Garden. Meanwhile, in terms of psychological experience perception indicators, comfort emerged as the most prominent factor influencing visitors’ garden experience, with aesthetic appeal ranking second.

**Table 2:** Principal component analysis of 12 perception indicators.

Category	Principal Component	Eigenvalue	Contribution rates/%	Accumulative contribution rate/%
Natural Perception Indicators	Uniqueness (E1)	2.81	0.42	0.42
	Vegetation richness (E2)	1.66	0.24	0.66
	Integration of carvings with the surrounding environment (E3)	0.79	0.12	0.78
	Architectural harmony with carvings(E4)	0.62	0.09	0.87
	Number of carvings (E5)	0.46	0.07	0.94
	Biodiversity(E6)	0.41	0.06	1
Psychological Perception Indicators	Comfort (E7)	1.55	0.48	0.48
	Aesthetic appeal (E8)	0.74	0.23	0.71
	Sense of security (E9)	0.37	0.12	0.83
	Artistic value (E10)	0.23	0.07	0.90
	Historical and educational value (E11)	0.19	0.06	0.96
	Attractiveness (E12)	0.14	0.04	1

To further explore the mechanisms through which these key elements impact visitor experiences, this study specifically focuses on the uniqueness of rock carvings within natural environment indicators and comfort within psychological experience perception indicators. Prior research has highlighted that the uniqueness is a critical criterion in the evaluation system of rock carving art, while in the field of landscape perception assessment, comfort has been recognized as a fundamental factor shaping overall visitor experience. Therefore, this study designates rock carving uniqueness and comfort as dependent variables to further investigate the relationships between these elements and visitor perceptions, as well as the underlying influencing factors.

### 4.3 Model performance evaluation and result analysis

Based on Principal Component Analysis (PCA), we selected “Uniqueness” as the most significant natural environment perception indicator and “Comfort” as the most influential psychological perception indicator to serve as the dependent variables in this study (Table 3). By employing multiple machine learning algorithms to explore the specific factors influencing psychological perception, we conducted an in-depth evaluation of model performance using confusion matrices to assess predictive accuracy. A total of 100 validation trials were conducted, and the results demonstrated that the Random Forest model outperformed others across three key evaluation metrics: Accuracy,  Macro F1−Score, and Weighted F1−Score.

**Table 3:** Performance of machine learning algorithms.

Perceived categories	Model	Linear	Non-linear	
	Performance	Logistic regression	Random forest	SVM
Uniqueness	Accuracy	0.53	0.73	0.67
	Macro F1-Score	0.44	0.68	0.64
	Weighted F1-Score	0.52	0.72	0.68
Comfort	Accuracy	0.53	0.67	0.47
	Macro F1-Score	0.37	0.73	0.33
	Weighted F1-Score	0.63	0.68	0.53

The confusion matrix is a table used to evaluate the performance of a classification model, showing the comparison between the predicted results and the actual labels [63]. Specifically, for “Uniqueness”, the confusion matrix analysis revealed that all four samples classified as −1 (negative category) were correctly predicted, while some misclassification was observed in the neutral (0) and positive (1) categories (Table 4). This indicates that the model has room for improvement in distinguishing category boundaries more accurately. Similarly, for “Comfort”, the confusion matrix highlighted a weaker performance in identifying negative (−1) categories, despite achieving relatively higher accuracy for the other categories.

**Table 4:** Confusion matrix for model predictions.

Metric	Actual/ Predicted	−1	0	1
Uniqueness	−1	**4**	0	0
	0	1	**1**	1
	1	2	0	**6**
Comfort	−1	**1**	0	0
	0	0	**2**	2
	1	0	3	**7**

Note: The experiment was conducted on a dataset of 96 samples, split into 81 training samples and 15 testing samples.

### 4.4 Influence of environmental factors on perceptual indicators

**Fig 3:**
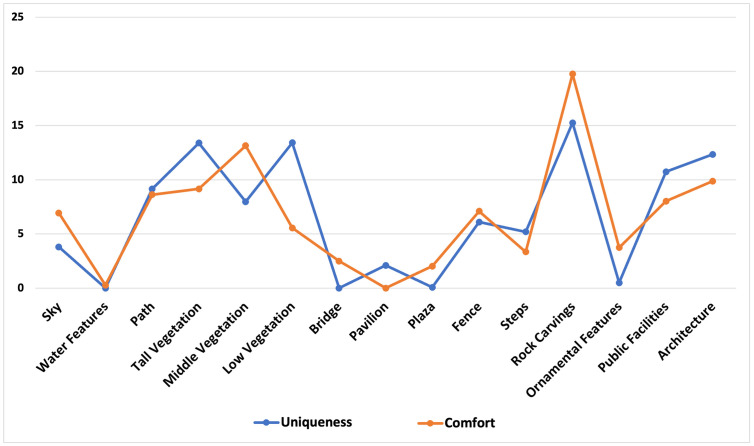
Relative importance of indicators calculated by the random forest model.

The results of the random forest analysis reveal significant differences in the relative importance of environmental factors in shaping visitors’ perceptual experiences (see Fig 3). Specifically, rock carvings, high-layer vegetation, middle vegetation, and low-layer vegetation exhibit higher importance scores within the model, indicating that these elements are key determinants of visitor perception.

Notably, rock carvings received the highest evaluation in both uniqueness and comfort, underscoring their prominent contribution to these two dimensions. Similarly, various types of vegetation demonstrated a comparably high value orientation, emphasizing the critical role of natural elements in enhancing visitor experiences. In contrast, elements such as plazas and pavilions did not exhibit a significant impact on visitors’ perceptual emotions, suggesting that their contribution to the overall experience may be relatively limited.

**Fig 4:**
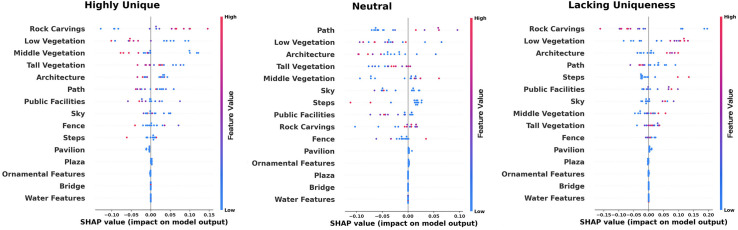
Relative importance of different environmental elements in perceived uniqueness.

Although the relative importance analysis helps clarify the significance of various indicators, a more precise understanding of their specific relationships with perceptual experience was achieved using SHAP value plots. By leveraging the random forest model, we examined the contribution of different feature values. As shown in Fig 4, the SHAP value plots illustrate the impact of environmental features on the uniqueness perception across different rating levels: excellent, moderate, and good.

Among the environmental features influencing model outputs, low vegetation and path consistently exhibited a significant positive impact, indicating their strong contribution to increasing the model’s output probability. In comparison, rock carvings also demonstrated a notable positive effect, though their influence was lower than that of low vegetation and path in the “neutral” category. The effects of architectural elements, tall vegetation, and middle vegetation were relatively neutral, suggesting a minimal impact on the model’s output. Conversely, features such as the sky, steps, and other elements—including public facilities, fences, pavilions, ornamental features, plazas, bridges, and water—displayed either minimal or even negative effects in different analyses.

**Fig 5:**
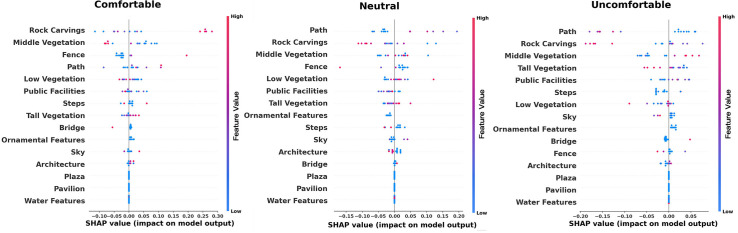
Relative importance of different environmental elements in perceived comfort.

Fig 5 presents the ranking of relative importance for different elements in relation to comfort levels categorized as comfortable, neutral, and uncomfortable. Through the analysis of SHAP values across multiple visualizations, professional insights into the impact of various environmental features on model outputs can be summarized. Path and low vegetation consistently exhibit a significant positive influence, indicating their crucial role in enhancing model prediction outcomes. Rock carvings also demonstrate a strong positive effect, with their influence being particularly prominent. In contrast, middle vegetation, the sky, and steps show a mild to moderate positive impact on model outputs. Notably, buildings, tall vegetation, and public facilities have a relatively neutral effect, suggesting their limited contribution to the model’s predictions. On the other hand, elements such as fences, bridges, plazas, ornamental features, pavilions, and water generally display a minor or even negative influence, implying that these features may slightly decrease the probability of higher model output values.

### 4.5 Comparison of logistic regression and random forest

Table 5 presents the results of the random forest relative importance analysis and Pearson correlation coefficients, aiming to assess the impact of different environmental elements on perceived uniqueness and comfort experience. In the evaluation of perceived uniqueness, the rock carvings element exhibited the highest relative importance (15.25), and its correlation showed a significant positive relationship (r = 0.46^***^, p < 0.001). Additionally, although low vegetation and tall vegetation elements also displayed relatively high relative importance (13.41,13.39), they demonstrated a negative correlation with perceived uniqueness (r = −0.328^**^, p < 0.01; r = −0.235^*^, p < 0.05).

**Table 5:** Comparison of results between random forest and pearson correlation coefficients.

Element	Uniqueness		Comfort	
	Random forest	Pearson correlation	Random forest	Pearson correlation
Sky	3.8	−0.072	6.93	0.005
Water Features	0	−0.073	0.27	0.09^*^
Path	9.16	−0.145	8.63	0.066
Tall Vegetation	13.39	−0.235^*^	9.15	0.033
Middle Vegetation	7.97	−0.167	13.14	−0.178^**^
Low Vegetation	13.41	−0.328^**^	5.55	−0.07^*^
Bridge	0	0.153	2.49	−0.095^*^
Pavilion	2.1	−0.102	0	0.081^*^
Plaza	0.07	−0.157	2.02	0.109^*^
Fence	6.1	0.018	7.1	0.238^**^
Steps	5.19	−0.096	3.34	0.121^*^
Rock Carvings	15.25	0.46^***^	19.75	−0.014
Ornamental Features	0.5	0.143	3.74	0.131^*^
Public Facilities	10.73	−0.174	8.02	−0.019
Architecture	12.34	−0.08	9.87	−0.084^*^

Note:

*** p < 0.001,

** p < 0.01,

* p < 0.05

For perceived comfort, although the rock carvings element had the highest relative importance in the random forest model (19.75), the correlation analysis did not show a significant effect on comfort. In contrast, the relatively lower relative importance of the fence element demonstrated a significant positive correlation (r = 0.238^**^, p < 0.01). Furthermore, although the middle vegetation element had the second-highest relative importance (13.14), it exhibited a negative correlation with comfort (r = −0.178^**^, p < 0.01). Notably, factors that demonstrated a positive correlation with comfort, such as water, pavilions, and steps, despite ranking lower in relative importance, still exhibited positive correlations (r = 0.09^*^, p < 0.05; r = 0.081^*^, p < 0.05; r = 0.109^*^, p < 0.05), indicating that even elements with lower importance ratings can still make a positive contribution to an individual’s comfort experience.

## 5. Discussion

### 5.1 Changes in tourists’ perception of uniqueness

This study, from the perspective of visitor perception, conducts an empirical analysis of the rock carving art experience in Wild Mountain Spring and Autumn Garden, systematically revealing the multidimensional interactive relationship between environmental elements and visitors’ psychological perceptions. The findings indicate that rock carvings, as the core attraction of the park, rank first in perceived “uniqueness” among natural environment perception indicators. Their irreplaceable artistic value, cultural symbolism, and spatial characteristics stimulate visitors’ curiosity and desire for exploration, making them the key drivers of the experience. This finding echoes Christian Norberg-Schulz’s theory of “Genius Loci,” which posits that spaces bearing unique historical memories or geographical markers can foster deep emotional connections between people and their environment, specifically manifested in enhanced sense of belonging and identity formation [64]. Notably, although the hierarchical vegetation system composed of tall trees and low shrubs dominates the visual landscape, it shows a significant negative correlation with visitors’ perception of uniqueness. This not only validates the mechanism by which natural elements influence psychological health in ecological psychology [65], but also reveals room for optimization in the current vegetation system’s role in shaping landscape distinctiveness. Further analysis shows that multi-layered vegetation configuration is among the top five factors influencing visitor experience, and the three-dimensional arrangement of plant communities plays a crucial role in enhancing the quality of landscape perception. Based on this, the study suggests that while strengthening the core attraction of rock carving art, the vertical structure of trees, shrubs, and ground cover plants should be optimized to create plant spaces that are both biodiverse and aligned with cultural landscape characteristics, thereby forming a synergistic enhancement mechanism between rock art and the ecological environment. This optimization path not only continues the explicit role of vegetation in comfort perception as described in traditional linear models, but also, through the reconstruction of spatial topological relationships, activates the implicit perceptual value of vegetation elements, providing a new theoretical perspective for the ecological design of cultural heritage sites.

### 5.2 Changes in tourists’ perception of comfort in rock carvings

Through multidimensional experience perception metrics, the study finds that comfort triggers a deep psychological resonance and immersive perception state in tourists during their experience at Yeshan Spring and Autumn Garden. The research reveals the unique “dual effect” of middle vegetation in influencing tourists’ comfort perception: on one hand, its high weight in the random forest model and its significant positive predictive ability, combined with the statistical significance of the Pearson correlation coefficient, validate the positive role of shrub vegetation in psychological adjustment for tourists. This finding empirically supports the argument in cultural landscape theory that “micro-level vegetation intervention enhances perception quality” [66]. This mechanism can be explained from the perspective of ecological psychology: based on the Stress Recovery Theory (SRT), Ulrich’s (1983) empirical research shows that natural landscapes with visual accessibility (such as depth of field) and low-threat characteristics (such as curvilinear forms) can effectively reduce cortisol levels and promote autonomic nervous system balance [67]. In addition, the Attention Restoration Theory (ART) further points out that natural elements (such as vegetation) can significantly enhance individuals’ psychological restoration by attracting attention and reducing cognitive fatigue [68]. For example, Hartig et al. (2003) found that natural environments, compared to urban environments, are more effective in promoting mental health [69], while Staats et al. (2007), by comparing visual stimuli from forests and urban landscapes, confirmed the significant effect of natural environments in alleviating psychological stress [70].

On the other hand, while rock carving art did not show significant correlation in the traditional linear model, its dominant position in the nonlinear model suggests a potential impact pathway of cultural symbols on tourists’ cognition—likely triggered through implicit dimensions such as landscape narrative and historical associations, which evoke deep emotional resonance. This finding extends the existing explanation framework of the “explicit-implicit” factor synergy in current research.

### 5.3 Synergistic effects of garden elements and the mechanism of perception experience

This study finds that the synergistic effects of garden elements exhibit a nonlinear interaction mechanism in tourists’ perception experiences, with rock carving elements occupying the central position in driving perceptions of both uniqueness and comfort, while the vegetation system supplements these effects through its multilayered structure. This confirms the perception enhancement effect of ecological elements on core attractions in cultural heritage sites. It is important to note that the element action mechanism reveals a significant “paradigm difference” in analysis: the Pearson linear model shows that fence elements and hydrological landscapes directly influence comfort perception, aligning with studies on water landscape perception in urban parks [71,72]. In contrast, the random forest nonlinear model reveals that lower-weight elements, such as ornamental features and bridges, generate perceptual transition effects through spatial sequencing. This distinction between “explicit-implicit” effects suggests that the formation of tourist experiences relies not only on the physical attributes of elements but also on the spatial topological relationships between elements—for example, fences enhance the ritualistic sense of rock carving viewing through visual guidance, while water elements subtly adjust the environmental atmosphere through soundscapes. Based on these insights, the study proposes a “dual-dimensional optimization framework”: at the element configuration level, priority should be given to ensuring the visibility of rock carvings and the richness of vegetation layers, while at the spatial organization level, a narrative connection through bridges, corridors, and fences should be used to activate the catalytic perception effect of secondary elements.

### 5.4 Practical significance and future research

This study aims to investigate the application and significance of optimizing the surrounding environment of rock carvings and enhancing tourists’ cultural experience and perception in modern gardens. The results indicate that rock carving elements are key to enhancing landscape appeal, especially in areas with weak visual perception. The scientific configuration of middle vegetation can significantly stimulate tourists’ curiosity and desire to exploration. However, it is important to note that the sample size in this study is relatively small and limited to specific regions, which restricts the generalizability of the findings. Consequently, the data collected may not fully capture the diversity of tourists’ perceptual experiences across different areas. It is worth noting that the sample size is relatively small and confined to specific regions, which restricts the generalizability of the conclusions, Therefore, it is recommended that a “one garden, one policy” strategy be adopted for rock carving gardens in various geographical locations. This approach entails developing tailored design schemes based on the unique environmental, cultural, and historical contexts of each garden. To achieve this, a comprehensive and systematic evaluation indicator system should be established to ensure that design plans accurately address local characteristics and needs, thereby optimizing both the protection and development of the gardens.

In this study, the random forest algorithm was preliminarily introduced to explore the nonlinear relationships between various elements in rock carving gardens and tourists’ perceptions, revealing some potential influencing patterns. While machine learning was employed to investigate these nonlinear relationships, the analysis of the directionality and dynamic processes of interactions remains insufficient. To further elucidate these complex relationships—particularly the existence and mechanisms of threshold effects—future research should conduct more in-depth investigations. Through systematic analysis and modeling, such efforts will not only provide theoretical support for the design of rock carving gardens but also effectively advance the dual objectives of cultural heritage preservation and environmental protection.

## 6. Conclusion

This study employs an interdisciplinary approach to deeply analyze the synergistic interaction mechanisms between cultural heritage sites, rock carvings, and landscape elements, as well as their impact on visitors’ perceptual experiences. The main conclusions drawn from the study are as follows: (1) Uniqueness and comfort together form the core dimensions of visitors’ perceptions, with rock carvings being the key anchor point for the perceptual experience. (2) In optimizing visitor perception, the configuration of middle vegetation, particularly in spatial permeability and morphological control, is especially important, and it shows the best benefits in perception evaluation. (3) The study preliminarily reveals the nonlinear relationship between environmental elements and visitors’ perceptions, pointing out the limitations of traditional linear analysis methods. (4) Based on the unique characteristics of rock carving gardens in different regions, the study proposes a “one garden, one strategy” design concept, emphasizing the need to tailor protection and development strategies according to local natural conditions, historical and cultural backgrounds, and social needs.

Future research should focus on deepening multimodal perceptual analysis, combining eye-tracking and skin response monitoring technologies, to quantify subconscious environmental response mechanisms. At the same time, it is recommended that future studies expand the sample size and include more diverse visitor groups to enhance the representativeness and applicability of the findings. This would help establish a more comprehensive evaluation system and optimization strategies that promote the synergistic development of cultural heritage conservation and ecological sustainability.

## Supporting information

S1 Appendix Survey on visitors’ perception. (DOCX)

S1 File Supplementary data. (DOCX)
