# Supplementary material for: Exploring visitor perception and environmental element relationships in rock carving landscapes through random forest analysis
Source: PLoS One. 2025 Jul 1;20(7):e0326302. doi: 10.1371/journal.pone.0326302 (PMC12212488; doi:10.1371/journal.pone.0326302)
Supplement: S1 Appendix — (DOCX) [file pone.0326302.s001.docx]

**Supporting information**

**S1 Appendix. A: Survey on visitors' perception.**


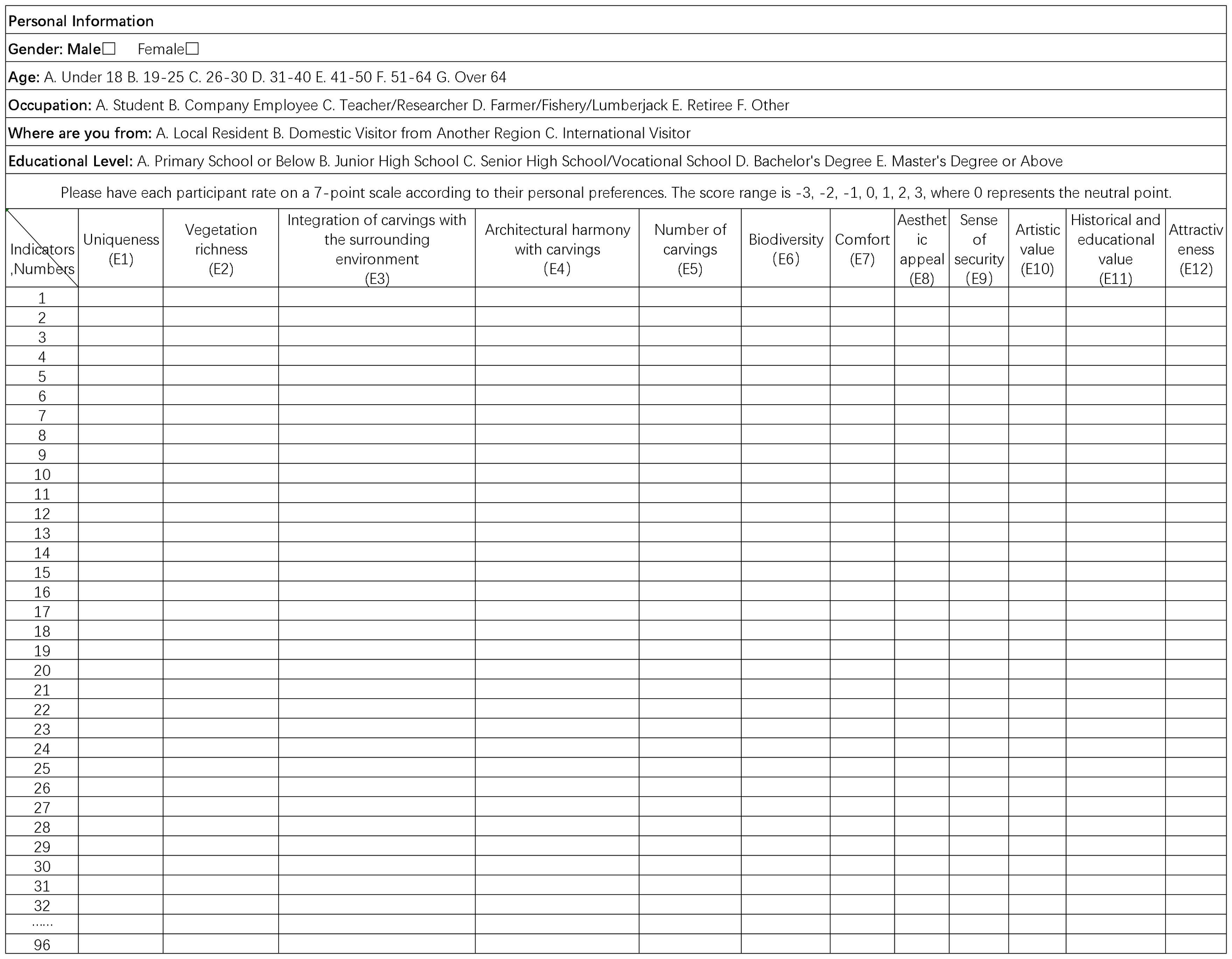
Questionnaire for field data collection on visitor perceptions.
