# Supplementary material for: Exploring visitor perception and environmental element relationships in rock carving landscapes through random forest analysis
Source: PLoS One. 2025 Jul 1;20(7):e0326302. doi: 10.1371/journal.pone.0326302 (PMC12212488; doi:10.1371/journal.pone.0326302)
Supplement: S1 File — (DOCX) [file pone.0326302.s002.docx]

**Supporting information**

**A.Source Data (Semantic Image Segmentation)**

| Image | Sky | Water Features | Path | Tall Vegetation | Middle Vegetation | Low Vegetation | Bridge | Pavilion | Plaza | Fence | Steps | Rock Carvings | Ornamental Features | Public Facilities | Architecture |
| --- | --- | --- | --- | --- | --- | --- | --- | --- | --- | --- | --- | --- | --- | --- | --- |
| 1 | 0.01685 | 0 | 0.099611 | 0.398015 | 0 | 0.179032 | 0 | 0 | 0 | 0 | 0 | 0.229764 | 0 | 0.008167 | 0.045847 |
| 2 | 0.14267 | 0 | 0.015716 | 0.431653 | 0 | 0.28605 | 0 | 0 | 0 | 0 | 0 | 0.034223 | 0 | 0 | 0.07687 |
| 3 | 0.026428 | 0 | 0.103742 | 0.054796 | 0 | 0.106785 | 0 | 0 | 0 | 0.087058 | 0 | 0.149334 | 0 | 0.030372 | 0.42734 |
| 4 | 0.005382 | 0 | 0.180556 | 0.557983 | 0.020254 | 0.004159 | 0 | 0 | 0 | 0.117901 | 0 | 0.073828 | 0 | 0 | 0.024702 |
| 5 | 0.04861 | 0 | 0.102005 | 0.539589 | 0.030388 | 0 | 0 | 0 | 0 | 0.215071 | 0 | 0.043654 | 0 | 0.008428 | 0 |
| 6 | 0.103839 | 0 | 0.037758 | 0.387164 | 0.153554 | 0 | 0 | 0 | 0 | 0.037828 | 0 | 0.057251 | 0 | 0.007645 | 0.160116 |
| 7 | 7.01E-05 | 0 | 0.099293 | 0.461684 | 0.078907 | 0 | 0 | 0 | 0 | 0 | 0 | 0.310553 | 0 | 0 | 0.039957 |
| 8 | 0 | 0 | 0.060972 | 0.560029 | 0.135563 | 0.000873 | 0 | 0 | 0 | 0 | 0.116387 | 0.024826 | 0 | 0.030113 | 0.025282 |
| 9 | 0.012927 | 0 | 0.037561 | 0.538724 | 0 | 0 | 0 | 0 | 0 | 0 | 0.011925 | 0.25716 | 0 | 0 | 0.016069 |
| 10 | 0.059632 | 0 | 0 | 0.255959 | 0 | 0 | 0 | 0 | 0 | 0 | 0 | 0.669795 | 0 | 0 | 0 |
| 11 | 0 | 0 | 0 | 0.425788 | 0 | 0 | 0 | 0 | 0 | 0 | 0 | 0.539472 | 0 | 0 | 0 |
| 12 | 0.011581 | 0 | 0.041019 | 0.650525 | 0.038402 | 0 | 0 | 0 | 0 | 0 | 0 | 0.154487 | 0 | 0 | 0 |
| 13 | 0 | 0 | 0 | 0 | 0 | 0 | 0 | 0 | 0 | 0 | 0 | 0.193651 | 0.80133 | 0 | 0 |
| 14 | 0 | 0 | 0.031591 | 0.692399 | 0.169513 | 0.022917 | 0 | 0 | 0 | 0 | 0 | 0.028051 | 0 | 0.005928 | 0.04258 |
| 15 | 0 | 0 | 0.02747 | 0.551447 | 0.082267 | 0.003076 | 0 | 0 | 0 | 0 | 0.013116 | 0.209157 | 0 | 0.004939 | 0 |
| 16 | 0 | 0 | 0.165416 | 0.471171 | 0.169669 | 0.030792 | 0 | 0 | 0 | 0 | 0 | 0.051658 | 0 | 0.010267 | 0.071564 |
| 17 | 0 | 0 | 0.077921 | 0.561915 | 0.038817 | 0.033108 | 0 | 0 | 0 | 0.00143 | 0 | 0.234844 | 0 | 0.007377 | 0.020807 |
| 18 | 0.119094 | 0 | 0.029039 | 0.210714 | 0.245817 | 0.102759 | 0 | 0 | 0 | 0 | 0.099181 | 0.012291 | 0 | 0.093983 | 0.011034 |
| 19 | 0.033916 | 0 | 0.027569 | 0.448596 | 0.213657 | 0.08306 | 0 | 0 | 0 | 0 | 0 | 0.144173 | 0 | 0.011139 | 0.018428 |
| 20 | 0 | 0 | 0.011785 | 0.360869 | 0.224364 | 0.06674 | 0 | 0 | 0 | 0 | 0 | 0.179933 | 0 | 0 | 0.02889 |
| 21 | 0.004592 | 0 | 0.104261 | 0.218224 | 0.198675 | 0.023948 | 0 | 0 | 0 | 0 | 0 | 0.412266 | 0 | 0 | 0 |
| 22 | 0 | 0 | 0 | 0.157984 | 0.384303 | 0.132368 | 0 | 0 | 0 | 0 | 0 | 0.293761 | 0 | 0.003642 | 0 |
| 23 | 0.000157 | 0 | 0 | 0.372142 | 0.154879 | 0.119611 | 0 | 0 | 0.026548 | 0 | 0.043326 | 0.247709 | 0 | 0.019164 | 0 |
| 24 | 0.023838 | 0 | 0.007392 | 0.400054 | 0.140552 | 0.132281 | 0 | 0 | 0.106268 | 0 | 0.03913 | 0.080991 | 0 | 0.003301 | 0 |
| 25 | 0.014823 | 0 | 0.032396 | 0.364519 | 0.172652 | 0.165495 | 0 | 0 | 0.09016 | 0 | 0.005542 | 0.055209 | 0 | 0.006442 | 0.034635 |
| 26 | 0 | 0 | 0.04214 | 0.478443 | 0.135637 | 0.145883 | 0 | 0 | 0 | 0 | 0 | 0.172663 | 0 | 0 | 0 |
| 27 | 0.00016 | 0 | 0.19541 | 0.521034 | 0.034786 | 0.027271 | 0 | 0 | 0 | 0 | 0.040696 | 0.141204 | 0 | 0.001604 | 0 |
| 28 | 0.051174 | 0 | 0.099101 | 0.511018 | 0.167721 | 0.06301 | 0 | 0 | 0 | 0 | 0.008349 | 0 | 0 | 0.013257 | 0.040418 |
| 29 | 0.036244 | 0 | 0 | 0 | 0.511079 | 0.058892 | 0 | 0 | 0 | 0 | 0 | 0.238439 | 0 | 0.003084 | 0 |
| 30 | 0.006178 | 0 | 0.218026 | 0.386099 | 0.009023 | 0.054391 | 0 | 0 | 0 | 0 | 0 | 0.041177 | 0 | 0.022283 | 0.02163 |
| 31 | 0.003201 | 0 | 0.398271 | 0.299675 | 0.009204 | 0.052951 | 0 | 0 | 0 | 0 | 0 | 0 | 0 | 0.056411 | 0 |
| 32 | 0.182628 | 0 | 0.001889 | 0.266374 | 0.03879 | 0.250999 | 0 | 0 | 0 | 0 | 0.015097 | 0.021349 | 0 | 0.010181 | 0.112564 |
| 33 | 0.00499 | 0 | 0.013629 | 0.22314 | 0.074678 | 0.200983 | 0 | 0 | 0 | 0 | 0.052488 | 0.376622 | 0 | 0 | 0.00993 |
| 34 | 0.000111 | 0 | 0 | 0.298028 | 0.21063 | 0.03252 | 0 | 0 | 0 | 0 | 0 | 0.397795 | 0 | 0 | 0 |
| 35 | 0 | 0 | 0.075955 | 0.600301 | 0.034565 | 0.228253 | 0 | 0 | 0 | 0 | 0 | 0.031093 | 0 | 0 | 0 |
| 36 | 0.088262 | 0 | 0.105976 | 0.471455 | 0.015792 | 0.297544 | 0 | 0 | 0 | 0 | 0 | 0.004531 | 0 | 0.003841 | 0 |
| 37 | 0 | 0 | 0.076266 | 0.570965 | 0.02943 | 0.281212 | 0 | 0 | 0 | 0 | 0 | 0.036456 | 0 | 0 | 0 |
| 38 | 0 | 0 | 0.063125 | 0.60921 | 0.016557 | 0.243969 | 0 | 0 | 0 | 0 | 0 | 0.001763 | 0.002986 | 0.010401 | 0.031155 |
| 39 | 8.5E-06 | 0 | 0.201637 | 0.448949 | 0.010358 | 0.246997 | 0 | 0 | 0 | 0 | 0 | 0.001178 | 0 | 0 | 0.047377 |
| 40 | 0.000247 | 0 | 0.130727 | 0.633974 | 0.000205 | 0.146735 | 0 | 0 | 0 | 0 | 0 | 0.008138 | 0.026348 | 0.004483 | 0.037441 |
| 41 | 0 | 0 | 0.272297 | 0.465571 | 0.000225 | 0.038209 | 0 | 0 | 0 | 0 | 0 | 0.001166 | 0 | 0.023125 | 0.061987 |
| 42 | 0 | 0 | 0.244542 | 0.246281 | 0.003843 | 0.06035 | 0 | 0 | 0 | 0 | 0 | 0.0021 | 0.000838 | 0.31598 | 0.000287 |
| 43 | 6.25E-06 | 0.00992 | 0.174839 | 0.52484 | 0.0052 | 0.138261 | 0 | 0 | 0 | 0.06142 | 0.000894 | 0 | 0 | 0 | 0.064951 |
| 44 | 0.000685 | 0 | 0.018726 | 0.711379 | 0.002174 | 0.089138 | 0 | 0 | 0 | 0 | 0.024312 | 0.024232 | 0 | 0.007585 | 0.108038 |
| 45 | 0 | 0 | 0.021778 | 0.273063 | 0.00173 | 0.359517 | 0 | 0 | 0 | 0.051605 | 0 | 0 | 0.032741 | 0.000402 | 0.251996 |
| 46 | 1.72E-06 | 0.236666 | 0 | 0.646599 | 0.001468 | 0 | 0 | 0 | 0 | 0.051975 | 0.047709 | 0 | 0 | 0 | 0 |
| 47 | 0.00012 | 0 | 0.138272 | 0.5323 | 0.019111 | 0.080185 | 0 | 0 | 0 | 0.173378 | 0.003781 | 0.000983 | 0 | 0 | 0.016105 |
| 48 | 0.031256 | 0 | 0.079758 | 0.254159 | 0.050537 | 0.101528 | 0 | 0 | 0 | 0.147361 | 0 | 0 | 0.08216 | 0.005202 | 0.202499 |
| 49 | 0 | 0 | 0.140392 | 0.599884 | 0.068244 | 0.105933 | 0 | 0.016949 | 0 | 0 | 0 | 0.003475 | 0 | 0.030753 | 0.001461 |
| 50 | 0.093478 | 0 | 0.036129 | 0.309962 | 0.006006 | 0.100724 | 0 | 0.298891 | 0 | 0 | 0.023228 | 0.016524 | 0 | 0.002912 | 0.084967 |
| 51 | 0 | 0 | 0.000106 | 0.540913 | 0.126225 | 0.204435 | 0 | 0 | 0 | 0 | 0 | 0.104091 | 0 | 0.001378 | 0 |
| 52 | 0 | 0 | 0.000639 | 0.613746 | 0.121214 | 0.16797 | 0.001738 | 0 | 0 | 0.005236 | 0 | 0.067319 | 0 | 0.003997 | 0.001747 |
| 53 | 0 | 0 | 0.023022 | 0.629338 | 0.131944 | 0.093725 | 0.010956 | 0 | 0 | 0.083466 | 0 | 0.000888 | 0 | 0 | 0.003226 |
| 54 | 0 | 0 | 0.000238 | 0.379955 | 0.192106 | 0.174157 | 0 | 0 | 0 | 0.052649 | 0.006566 | 0.181901 | 0 | 0 | 0 |
| 55 | 0.083392 | 0 | 0 | 0.256101 | 0.1337 | 0.101129 | 0 | 0 | 0 | 0 | 0.05257 | 0.25558 | 0 | 0 | 0 |
| 56 | 0.105811 | 0 | 0.000457 | 0.464935 | 0.120133 | 0 | 0 | 0.059221 | 0 | 0.007233 | 0 | 0.2291 | 0 | 0 | 0.002791 |
| 57 | 0.048045 | 0 | 0 | 0.363273 | 0.145394 | 0.114393 | 0 | 0 | 0 | 0 | 0.046877 | 0.264956 | 0 | 0 | 0.001654 |
| 58 | 0.082217 | 0 | 0.028703 | 0.333494 | 0 | 0.156102 | 0 | 0 | 0 | 0.002517 | 0 | 0.303437 | 0.00146 | 0 | 0.082588 |
| 59 | 0.053965 | 0 | 0.031221 | 0.636301 | 0.002874 | 0.143023 | 0 | 0 | 0 | 0.030191 | 0 | 0.064672 | 0.001819 | 0 | 0.018519 |
| 60 | 0.019964 | 0 | 0.090973 | 0.583911 | 0.025882 | 0.117419 | 0 | 0 | 0 | 0.095605 | 0 | 0.016598 | 0 | 0.008042 | 0.00214 |
| 61 | 1.23E-07 | 0 | 0.028272 | 0.514218 | 0.023951 | 0.130485 | 0 | 0 | 0 | 0.164734 | 0 | 0.071913 | 0 | 0 | 0.002271 |
| 62 | 0.028056 | 0 | 0.027442 | 0.237663 | 0.04392 | 0.109501 | 0 | 0.0213 | 0 | 0.132715 | 0 | 0.001661 | 0.351453 | 0.000716 | 0.026297 |
| 63 | 0 | 0 | 0.01702 | 0.512549 | 0.131865 | 0.015592 | 0 | 0 | 0 | 0 | 0 | 0.311119 | 0 | 0 | 0 |
| 64 | 0.026004 | 0 | 0.270987 | 0.203507 | 0.03582 | 0.036303 | 0 | 0 | 0 | 0.058419 | 0.009304 | 0.267725 | 0 | 0.00218 | 0.07658 |
| 65 | 0 | 0 | 0.033238 | 0.192728 | 0 | 0.151188 | 0 | 0 | 0 | 0 | 0 | 0.571616 | 0 | 0.007893 | 0 |
| 66 | 0.006283 | 0 | 0.029284 | 0.168966 | 0.099602 | 0.179007 | 0 | 0 | 0 | 0.01427 | 0.029211 | 0.413433 | 0 | 0.000791 | 0.050394 |
| 67 | 0 | 0 | 0.135156 | 0.473099 | 0.116603 | 0.080677 | 0 | 0 | 0 | 0 | 0 | 0.151367 | 0 | 0.015861 | 0.016605 |
| 68 | 0 | 0 | 0 | 0.228635 | 0 | 3.62E-05 | 0 | 0 | 0 | 0 | 0 | 0.767963 | 0 | 0 | 0 |
| 69 | 0 | 0 | 0 | 0 | 0.043903 | 0.084506 | 0 | 0 | 0 | 0 | 0 | 0.869626 | 0 | 0 | 0 |
| 70 | 0 | 0 | 0 | 0.250818 | 0 | 0 | 0 | 0 | 0 | 0 | 0 | 0.748412 | 0 | 0 | 0 |
| 71 | 0 | 0 | 0.135041 | 0.491282 | 0.015436 | 0.040379 | 0 | 0 | 0 | 0.002997 | 0 | 0.295801 | 0 | 0 | 0.001818 |
| 72 | 0 | 0 | 0.124434 | 0.51346 | 0.05344 | 0.065065 | 0 | 0 | 0 | 0.021245 | 0 | 0.180677 | 0 | 0 | 0 |
| 73 | 4.09E-07 | 0 | 0 | 0.452381 | 0.062029 | 0 | 0 | 0 | 0 | 0 | 0.010095 | 0.46508 | 0 | 0 | 0 |
| 74 | 0.013917 | 0 | 0.14275 | 0.368802 | 0.057735 | 0.015264 | 0.007826 | 0 | 0 | 0.003311 | 0 | 0.29612 | 0 | 0.004489 | 0.065707 |
| 75 | 0 | 0 | 0 | 0.181543 | 0 | 0 | 0 | 0 | 0 | 0 | 0 | 0.80768 | 0 | 0.009518 | 0 |
| 76 | 0 | 0 | 0.025934 | 0.29713 | 0 | 0 | 0 | 0 | 0 | 0 | 0 | 0.588479 | 0 | 0.007733 | 0 |
| 77 | 0 | 0 | 0.013117 | 0.421676 | 0.02959 | 0.002084 | 0 | 0 | 0 | 0.005488 | 0.004585 | 0.500428 | 0 | 0 | 0 |
| 78 | 0.009731 | 0 | 0.062956 | 0.098192 | 0.022974 | 0.025916 | 0 | 0 | 0 | 0 | 0 | 0.678807 | 0 | 0.011094 | 0.088215 |
| 79 | 0.001289 | 0 | 0.034863 | 0.328337 | 0.118652 | 0.13396 | 0 | 0 | 0 | 0.02515 | 0.102552 | 0.233691 | 0 | 0 | 0 |
| 80 | 0 | 0 | 0 | 0.344191 | 0.172093 | 0.097805 | 0 | 0 | 0 | 0 | 0.153491 | 0.216019 | 0 | 0.005327 | 0 |
| 81 | 0 | 0.046692 | 0 | 0.203123 | 0.226627 | 0.013313 | 0 | 0 | 0 | 0 | 0 | 0.504721 | 0 | 0 | 0 |
| 82 | 0 | 0 | 0 | 0.293265 | 0 | 0 | 0 | 0 | 0 | 0 | 0 | 0.70639 | 0 | 0 | 0 |
| 83 | 0 | 0 | 0 | 0.177327 | 0 | 0 | 0 | 0 | 0 | 0 | 0 | 0.822409 | 0 | 0 | 0 |
| 84 | 0 | 0 | 0 | 0.601126 | 0.094287 | 0 | 0 | 0 | 0 | 0 | 0 | 0.304565 | 0 | 0 | 0 |
| 85 | 0 | 0 | 0 | 0.487244 | 0.26934 | 0 | 0 | 0 | 0 | 0 | 0 | 0.237975 | 0 | 0 | 0 |
| 86 | 0 | 0 | 0 | 0.046412 | 0.075512 | 0 | 0 | 0 | 0 | 0.045801 | 0 | 0.711373 | 0 | 0 | 0.11546 |
| 87 | 0 | 0 | 0 | 0.436204 | 0.027693 | 0 | 0.522325 | 0 | 0 | 0 | 0 | 0 | 0 | 0.004542 | 0 |
| 88 | 0.027294 | 0.00917 | 0.005175 | 0.240761 | 0.342734 | 0.028034 | 0.17194 | 0.027531 | 0 | 0.035278 | 0 | 0.003168 | 0 | 0 | 0.078925 |
| 89 | 0 | 0 | 0.000384 | 0.466352 | 0.273304 | 0 | 0 | 0 | 0 | 0.007289 | 0.1439 | 0.084643 | 0 | 0 | 0.010032 |
| 90 | 0 | 0 | 0 | 0.499589 | 0.17044 | 0.187354 | 0 | 0 | 0 | 0 | 0 | 0.131214 | 0 | 0 | 0 |
| 91 | 0 | 0 | 0 | 0.325839 | 0.330015 | 0.091676 | 0 | 0 | 0 | 0 | 0.069495 | 0.174703 | 0 | 0.003316 | 0 |
| 92 | 0 | 0 | 0.268279 | 0.319356 | 0.001709 | 0.212951 | 0 | 0 | 0 | 0 | 0.115132 | 0.012223 | 0 | 0.041728 | 0.003427 |
| 93 | 0.183698 | 0 | 0.013425 | 0.18442 | 0.016241 | 0.294179 | 0 | 0 | 0 | 0.001782 | 0 | 0.229614 | 0.017413 | 0 | 0.043079 |
| 94 | 0.199079 | 0 | 0.128718 | 0.149398 | 0.002293 | 0.007659 | 0 | 0 | 0 | 0.044934 | 0.093659 | 0 | 0.004537 | 0 | 0.361036 |
| 95 | 0 | 0 | 0.006281 | 0.236835 | 0.125768 | 0.16884 | 0 | 0 | 0 | 0.036178 | 0.137231 | 0.257818 | 0 | 0 | 0.007391 |
| 96 | 0.018865 | 0 | 0 | 0.279036 | 0.257851 | 0.016061 | 0 | 0 | 0 | 0 | 0 | 0.416432 | 0 | 0 | 0 |

**B. Source Data (Visitor Perceptions)**

| Image | Uniqueness(E1) | Vegetation richness(E2) | Integration of carvings with the surrounding environment(E3) | Architectural harmony with carvings (E4) | Number of carvings(E5) | Biodiversity(E6) | Comfort(E7) | Aesthetic appeal(E8) | Sense of security(E9) | Artistic value(E10) | Historical and educational value(E11) | Attractiveness(E12) |
| --- | --- | --- | --- | --- | --- | --- | --- | --- | --- | --- | --- | --- |
| 1 | 1 | 1 | 1 | 0 | 1 | -1 | 0 | 0 | -1 | 1 | 1 | 1 |
| 2 | 1 | -1 | -1 | 0 | -1 | -1 | -1 | -1 | 0 | -1 | -1 | 0 |
| 3 | 0 | -1 | 0 | 0 | 1 | -1 | 0 | 0 | 0 | -1 | 1 | 1 |
| 4 | 1 | 0 | 1 | 0 | 0 | -1 | 1 | 1 | 1 | 1 | 0 | 1 |
| 5 | 1 | 0 | 0 | 1 | -1 | -1 | 1 | 1 | 1 | 1 | 0 | 1 |
| 6 | 1 | -1 | 1 | 1 | 0 | -1 | 0 | 1 | 0 | 1 | 0 | 0 |
| 7 | 1 | 0 | 1 | 1 | 1 | -1 | 1 | 1 | 0 | 0 | 0 | 0 |
| 8 | 1 | -1 | 1 | 0 | 0 | -1 | 1 | 1 | 1 | 1 | 0 | 1 |
| 9 | 0 | 1 | 1 | 1 | 1 | -1 | -1 | -1 | -1 | -1 | -1 | -1 |
| 10 | 0 | 1 | -1 | -1 | 1 | -1 | -1 | -1 | -1 | -1 | 1 | -1 |
| 11 | 1 | 1 | 1 | -1 | 1 | -1 | -1 | -1 | -1 | -1 | 0 | -1 |
| 12 | 1 | 1 | 1 | 0 | 1 | -1 | 0 | 0 | -1 | -1 | 0 | -1 |
| 13 | -1 | 1 | 0 | 0 | 0 | -1 | 1 | 1 | 0 | 1 | 1 | 1 |
| 14 | 1 | -1 | -1 | -1 | -1 | -1 | -1 | 0 | 0 | 0 | -1 | -1 |
| 15 | 1 | 1 | 1 | -1 | 1 | -1 | -1 | -1 | -1 | -1 | 1 | 0 |
| 16 | 1 | -1 | -1 | -1 | -1 | -1 | 0 | 0 | 1 | 0 | -1 | 0 |
| 17 | 1 | 0 | 0 | 0 | 0 | -1 | 1 | 0 | 1 | 1 | 0 | 1 |
| 18 | 1 | -1 | 1 | 1 | -1 | -1 | 1 | 1 | 1 | 1 | -1 | 1 |
| 19 | 1 | -1 | -1 | -1 | -1 | -1 | 1 | 1 | 0 | 0 | -1 | 0 |
| 20 | 1 | 0 | 1 | 0 | 0 | -1 | -1 | 0 | -1 | -1 | -1 | -1 |
| 21 | 1 | 0 | -1 | -1 | -1 | -1 | -1 | -1 | -1 | -1 | -1 | -1 |
| 22 | 1 | 1 | 1 | -1 | 0 | -1 | -1 | 1 | -1 | 0 | -1 | 0 |
| 23 | 1 | -1 | -1 | -1 | -1 | -1 | -1 | -1 | -1 | -1 | -1 | -1 |
| 24 | 1 | -1 | -1 | -1 | -1 | -1 | 1 | 0 | 0 | 1 | -1 | 1 |
| 25 | 1 | -1 | -1 | 0 | -1 | -1 | 1 | 1 | 0 | 1 | -1 | 1 |
| 26 | 1 | -1 | -1 | 0 | -1 | -1 | 1 | 1 | -1 | 1 | -1 | 1 |
| 27 | 1 | 1 | 1 | 1 | 1 | -1 | 1 | 1 | 1 | 1 | 1 | 1 |
| 28 | 0 | -1 | -1 | 0 | -1 | -1 | 0 | 0 | 0 | 0 | 0 | -1 |
| 29 | 1 | -1 | -1 | -1 | -1 | -1 | 0 | 0 | 0 | 0 | -1 | -1 |
| 30 | 0 | -1 | 0 | 0 | -1 | -1 | 1 | 0 | 1 | -1 | 1 | 0 |
| 31 | 1 | -1 | -1 | -1 | -1 | -1 | 0 | 0 | 0 | 0 | 1 | 0 |
| 32 | 1 | 1 | 1 | 1 | 0 | 0 | 1 | 0 | 1 | 0 | 0 | 0 |
| 33 | 1 | -1 | 0 | 0 | -1 | -1 | 0 | 0 | -1 | 0 | -1 | -1 |
| 34 | 1 | 0 | 1 | 0 | -1 | -1 | 0 | 0 | 0 | 0 | -1 | 0 |
| 35 | 1 | -1 | 0 | 0 | -1 | -1 | 1 | 1 | 0 | 0 | 0 | 0 |
| 36 | 1 | -1 | 0 | -1 | -1 | -1 | 0 | 1 | 1 | 0 | 0 | 0 |
| 37 | 1 | -1 | 0 | -1 | -1 | -1 | 0 | 0 | 0 | 0 | 0 | -1 |
| 38 | 1 | -1 | -1 | -1 | -1 | -1 | 0 | 0 | 0 | 0 | -1 | -1 |
| 39 | 1 | -1 | -1 | -1 | -1 | -1 | 0 | 0 | -1 | -1 | 1 | 0 |
| 40 | 1 | -1 | -1 | 0 | -1 | -1 | 0 | 0 | 0 | 0 | 1 | 0 |
| 41 | 0 | -1 | 0 | 0 | -1 | -1 | 0 | 0 | 0 | 0 | 1 | 0 |
| 42 | 0 | -1 | 0 | -1 | -1 | -1 | 0 | 0 | 0 | 0 | 1 | 0 |
| 43 | 1 | -1 | -1 | -1 | -1 | -1 | 1 | 0 | 0 | 0 | 0 | 0 |
| 44 | 1 | -1 | -1 | -1 | -1 | -1 | 0 | 0 | 0 | 0 | -1 | -1 |
| 45 | 0 | -1 | -1 | 0 | -1 | -1 | 0 | 0 | 0 | 1 | -1 | -1 |
| 46 | 1 | -1 | -1 | -1 | -1 | -1 | 1 | 1 | 0 | 0 | 0 | 1 |
| 47 | 1 | -1 | -1 | -1 | -1 | -1 | 1 | 1 | 0 | 0 | 0 | 1 |
| 48 | 1 | -1 | -1 | 0 | -1 | -1 | 0 | -1 | 0 | 1 | 1 | 0 |
| 49 | 1 | -1 | 0 | 1 | -1 | -1 | 0 | 0 | 1 | -1 | -1 | 0 |
| 50 | 0 | -1 | -1 | 0 | -1 | -1 | 1 | 0 | 0 | 0 | 0 | 0 |
| 51 | 1 | -1 | 1 | -1 | -1 | -1 | 1 | 0 | 0 | 0 | 0 | 0 |
| 52 | 1 | 0 | 0 | -1 | -1 | -1 | -1 | 0 | 0 | -1 | -1 | -1 |
| 53 | 1 | -1 | -1 | -1 | -1 | -1 | 1 | 1 | 1 | 1 | -1 | 1 |
| 54 | 1 | -1 | 0 | -1 | -1 | -1 | 0 | 0 | 0 | 0 | 0 | 0 |
| 55 | 1 | -1 | 1 | 0 | -1 | -1 | 0 | 0 | -1 | 0 | -1 | 0 |
| 56 | 1 | -1 | 0 | 0 | -1 | -1 | 0 | 1 | 0 | 1 | 0 | 1 |
| 57 | 1 | -1 | 1 | -1 | -1 | -1 | 0 | 0 | -1 | 0 | -1 | -1 |
| 58 | 0 | 1 | 0 | 1 | 1 | -1 | 0 | 1 | 0 | 1 | 1 | 1 |
| 59 | 0 | 1 | 1 | 1 | 1 | -1 | 1 | 1 | 0 | 1 | 1 | 1 |
| 60 | 1 | 1 | 1 | 0 | 0 | -1 | 1 | 0 | 0 | 1 | 1 | 1 |
| 61 | 1 | 1 | 1 | 1 | 0 | -1 | 1 | 1 | 0 | 1 | 1 | 1 |
| 62 | 0 | 1 | 0 | 0 | -1 | -1 | 1 | 1 | 0 | 1 | 1 | 1 |
| 63 | 1 | 1 | 1 | 0 | 1 | -1 | 0 | 0 | -1 | -1 | 1 | 0 |
| 64 | 0 | 1 | 1 | 1 | 1 | -1 | 1 | 1 | 0 | 1 | 1 | 1 |
| 65 | 0 | 1 | 1 | 0 | 1 | -1 | -1 | 0 | -1 | 1 | 1 | 1 |
| 66 | 0 | 1 | 1 | -1 | 1 | -1 | -1 | 0 | -1 | 0 | 1 | 1 |
| 67 | -1 | 1 | 1 | 1 | 1 | -1 | 1 | 1 | 1 | 0 | 1 | 1 |
| 68 | 0 | -1 | -1 | 0 | -1 | 0 | 1 | 1 | 0 | 0 | -1 | 1 |
| 69 | -1 | 1 | 0 | -1 | 0 | -1 | 1 | 1 | 0 | 1 | 1 | 1 |
| 70 | -1 | 1 | 1 | -1 | 1 | -1 | 0 | 1 | 0 | 1 | 1 | 1 |
| 71 | -1 | 0 | 1 | 1 | 0 | -1 | 1 | 0 | -1 | 0 | 1 | 0 |
| 72 | 0 | 1 | 1 | -1 | 0 | -1 | 0 | 0 | 1 | 0 | 1 | 1 |
| 73 | 0 | -1 | -1 | -1 | -1 | 0 | 1 | 1 | 1 | 0 | -1 | 0 |
| 74 | 0 | 0 | 1 | -1 | 0 | -1 | 0 | 1 | -1 | 1 | 1 | 0 |
| 75 | 0 | 1 | 1 | 1 | 0 | 0 | 1 | 0 | 1 | 0 | 1 | 1 |
| 76 | -1 | 1 | 0 | -1 | 1 | -1 | 1 | 1 | 1 | 1 | 1 | 1 |
| 77 | -1 | 1 | 1 | -1 | 1 | -1 | 1 | 1 | 1 | 1 | 1 | 0 |
| 78 | 0 | 1 | 1 | -1 | 0 | -1 | 0 | 0 | -1 | 1 | 1 | 1 |
| 79 | -1 | 1 | 1 | 1 | 1 | -1 | -1 | 0 | -1 | 0 | 1 | 1 |
| 80 | 0 | 1 | 1 | 0 | 0 | -1 | 1 | 1 | 1 | 0 | 0 | 1 |
| 81 | 1 | 1 | 0 | 0 | 1 | 0 | 0 | 0 | 1 | 1 | 1 | 0 |
| 82 | 0 | 1 | 1 | 0 | 1 | 0 | 1 | 1 | -1 | 1 | 1 | 1 |
| 83 | 0 | 1 | 1 | 0 | 1 | -1 | 1 | 1 | 1 | 1 | 1 | 1 |
| 84 | 0 | 0 | 1 | -1 | 0 | -1 | 0 | 0 | -1 | 1 | 1 | 0 |
| 85 | 0 | 1 | 0 | -1 | -1 | 0 | 0 | -1 | -1 | 0 | 1 | 0 |
| 86 | 0 | 1 | 1 | -1 | 1 | -1 | 1 | 1 | -1 | 1 | 1 | 0 |
| 87 | -1 | 1 | 1 | 1 | 0 | -1 | 0 | 1 | 0 | 1 | 1 | 1 |
| 88 | -1 | 1 | 1 | 0 | 0 | -1 | -1 | 0 | 0 | 0 | 1 | 0 |
| 89 | 0 | -1 | 1 | 0 | -1 | 0 | 1 | 1 | 0 | 1 | 0 | 1 |
| 90 | -1 | -1 | 0 | 0 | -1 | -1 | 0 | -1 | 0 | 0 | 0 | 0 |
| 91 | 0 | -1 | 0 | -1 | 0 | 0 | 1 | 1 | 0 | 1 | -1 | 0 |
| 92 | 1 | 0 | -1 | -1 | -1 | 1 | 0 | 1 | 0 | 1 | -1 | 0 |
| 93 | 0 | -1 | 0 | -1 | -1 | 0 | 1 | 0 | 0 | -1 | 0 | 0 |
| 94 | -1 | 1 | 1 | 0 | 1 | -1 | 0 | 0 | 0 | 1 | 1 | 0 |
| 95 | -1 | -1 | 0 | 0 | -1 | -1 | 1 | 1 | 0 | 0 | 1 | 1 |
| 96 | 0 | -1 | 0 | -1 | -1 | 0 | 0 | 0 | 0 | 1 | 0 | 1 |
